# The Fuzziness of the Molecular World and Its Perspectives

**DOI:** 10.3390/molecules23082074

**Published:** 2018-08-19

**Authors:** Pier Luigi Gentili

**Affiliations:** Dipartimento di Chimica, Biologia e Biotecnologie, Università di Perugia, Via Elce di sotto 8, 06123 Perugia, Italy; pierluigi.gentili@unipg.it; Tel.: +39-075-585-5573

**Keywords:** fuzzy logic, complexity, chemical artificial intelligence, human nervous system, fuzzy proteins, conformations, photochromic compounds, qubit

## Abstract

Scientists want to comprehend and control complex systems. Their success depends on the ability to face also the challenges of the corresponding computational complexity. A promising research line is artificial intelligence (AI). In AI, fuzzy logic plays a significant role because it is a suitable model of the human capability to compute with words, which is relevant when we make decisions in complex situations. The concept of fuzzy set pervades the natural information systems (NISs), such as living cells, the immune and the nervous systems. This paper describes the fuzziness of the NISs, in particular of the human nervous system. Moreover, it traces three pathways to process fuzzy logic by molecules and their assemblies. The fuzziness of the molecular world is useful for the development of the chemical artificial intelligence (CAI). CAI will help to face the challenges that regard both the natural and the computational complexity.

## 1. Introduction

**Figure 1:**
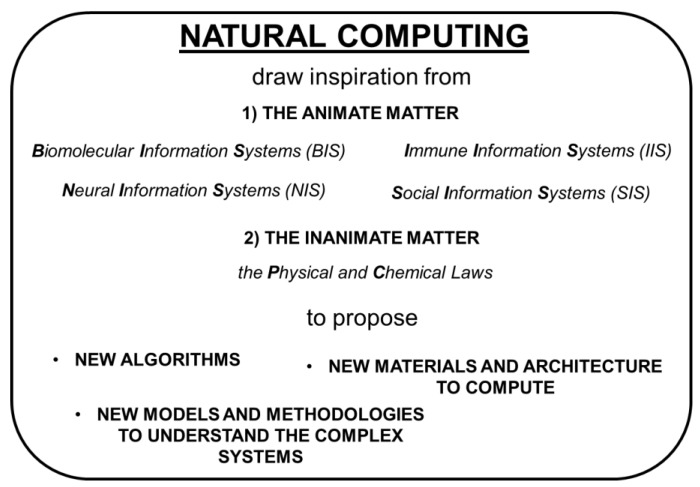
The contribution of the natural computing in coping with the challenges of the computational and natural complexity.

The scientific method, officially born in the seventieth century with the contributions of Galileo Galilei and Isaac Newton, has allowed humanity to become acquainted with the natural phenomena as never before. The acquisition of new scientific knowledge has also promoted an outstanding technological development in the last three hundred years or so. A mutual positive feedback relationship subsists between science and technology. To date amazing scientific and technological achievements have been reached. For example, we can explore the regions of the universe that are 10^26^ m far apart from us. At the same time, we can detect subatomic particles that have radii of the order of 10^−15^ m. We can record microscopic phenomena that occur in 10^−18^ s, but we can also retrieve traces of cosmic events happened billions of years ago. Our technology allows us to send robots to other planets of our solar system (e.g., the NASA Spirit rover on Mars), manipulate atoms and interfere with the expression of genes in living beings. Despite many efforts, there are still challenges that must be won. For instance, (I) we cannot predict catastrophic events on Earth (such as earthquakes and volcanic eruptions); (II) we strive to avoid the climate change; (III) we would like to exploit the energy and food resources without deteriorating the natural ecosystems and their biodiversity; (IV) there are diseases that are still incurable; (V) we would like to eradicate the poverty in the world; (VI) we make efforts to avoid or at least predict both economic and political crisis. Whenever we try to address such challenges, we experience frustrating insurmountable obstacles. Why? Because whenever we cope with one of them, we deal with a complex system. A complex system is one whose science is unable to give a complete and accurate description. In other words, scientists find difficulties in rationalizing and predicting the behaviors of complex systems. Examples of complex systems are the geology and the climate of the Earth; the ecosystems; each living being, in particular humans, giving rise to economic and social organizations, which are other examples of complex systems. The description of complex systems requires the collection, manipulation, and storage of big data [1], and the solution of problems of computational complexity. The description of complex systems from their ultimate constituents, i.e., atoms, is beyond our reach since the computational cost grows exponentially with the number of particles [2]. Moreover, many complex systems exhibit variable patterns. These variable patterns are objects (both inanimate and animate) or events whose recognition is made difficult by their multiple features, variability, and extreme sensitivity on the context. We still lack universally valid and effective algorithms for recognizing variable patterns [3]. Therefore, the obvious question is: How can we try to tackle the challenges regarding complex systems which involve issues of computational complexity? There are two principal strategies [4,5]. One consists in improving current electronic computers to make them faster and faster, and with increasingly large memory space. The other strategy is the interdisciplinary research line of natural computing. Researchers working on natural computing draw inspiration from Nature to propose: (I) new algorithms, (II) new materials and architecture for computing, and (III) new models to interpret complex systems. The sources of inspiration are the natural information systems, such as (a) the cells (i.e., the biomolecular information systems or BIS), (b) the nervous system (i.e., the neural information systems or NIS), (c) the immune system (i.e., the immune information systems or IIS), and (d) the societies (i.e., the societal information systems or SIS). Alternatively, we may exploit any causal event, involving inanimate matter, to make computation. In fact, in a causal event, the causes are the inputs and the effects are the outputs of a computation whose algorithm is defined by the laws governing the transformation (see Figure 1).

Among the natural information systems, the attention of many scientists worldwide is focused on the human nervous system that has human intelligence as its emergent property. The imitation of human intelligence is having a revolutionary impact in science, medicine, economy, security and well-being [6]. In fact, conventional quantitative techniques of system analysis are intrinsically unsuited for dealing with biological, social, economic, and any other type of system in which it is the behavior of the animate constituents that plays a dominant role. For such “humanistic systems”, the principle of incompatibility holds [7]: as the complexity of a system increases, our ability to make accurate and yet significant statements about its behavior diminishes until a threshold is reached beyond which accuracy and significance (or relevance) become almost mutually exclusive characteristics. An alternative approach is based on the human intelligence that has the remarkable power of handling both accurate and vague information. Information is vague when it is based on sensory perceptions. Vague information is coded through the words of our natural languages. Therefore, humans compute by using not only numbers but also and especially words. We have the remarkable capability to reason, speak, discuss and make rational decisions without any quantitative measurement and any numerical computation, in an environment of uncertainty, partiality, and relativity of truth. Moreover, we recognize quite easily variable patterns, such as human faces and voices. Therefore, a major challenge of the artificial intelligence research line is the comprehension and implementation of the capabilities of the human intelligence to compute with words [8]. The use of classical, Aristotelian, divalent logic implemented in electronic circuits and computers has allowed reproducing and even overcoming the human ability to compute with numbers. The imitation of human ability to compute with words is still challenging. Fuzzy logic is a good model. In fact, fuzzy logic has been defined as a rigorous logic of the vague and approximate reasoning [9]. In this paper, after describing the principal features of fuzzy logic, it is demonstrated that one reason why fuzzy logic is a valid model of the human power to compute with words can be found at the molecular level. Therefore, we propose the use of molecular, supramolecular, and chemical systems as an innovative strategy for implementing fuzzy logic. This article wants to pursue the idea of developing a chemical artificial intelligence [10], i.e., an artificial intelligence that is based not on electronic circuits and software, but on chemical reactions in a wetware. Probably, the chemical artificial intelligence will promote the design of a new generation of computational machines, more similar to the brain rather than to the electronic computers. These new brain-like “chemical computers” should help to cope with the challenges regarding the complex systems, aforementioned in this Introduction.

## 2. Some Features of Fuzzy Logic

**Figure 2:**
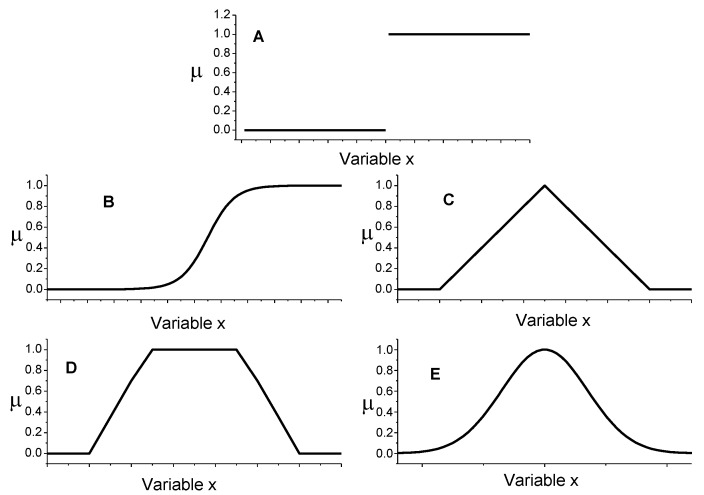
Shapes of the membership functions (μ) for a generic variable x: the case of a classical Boolean set in **A**; examples of fuzzy sets with sigmoidal, triangular, trapezoidal, and Gaussian shapes are shown in **B**, **C**, **D**, and **E** plots, respectively.

Fuzzy logic is based on the theory of fuzzy sets proposed by the engineer Lotfi Zadeh in 1965 [11]. A fuzzy set is different from a classical Boolean set. A classical set, also named as a crisp set, is a container that wholly includes or wholly excludes any given element. The theory of classical sets is based on the Law of Excluded Middle formulated by Aristotle in the fourth century BC. The Law of Excluded Middle states that an element x belongs to either set S or to its complement, i.e., set not-S. Zadeh proposed a refinement of the theory of the classical sets. In fact, a fuzzy set is more than a classical set: it can wholly include or wholly exclude elements, but it can also partially include and exclude other elements. The theory of fuzzy sets breaks the Law of Excluded Middle because an element x may belong to both set S and its complement not-S. An element x may belong to any set, but with different degrees of membership. The degree of membership (μ) of an element to a fuzzy set can be any real number included between 0 and 1. If μ=0, the element does not belong at all to the set; if μ=1, it completely belongs to the set; if 0<μ<1, the element belongs partially to the set. The Law of Excluded Middle is the foundation of the binary logic. In binary logic any variable is partitioned in two classical sets after fixing a threshold value: one set includes all the values below the threshold, whereas the other one contains those above. In the case of a positive logic convention, all the values of the first set become the binary 0, whereas those of the other set become the binary 1. The shape of a classical set is like that shown in Figure 2A. The degree of membership function for such a set discontinuously changes from 0 (below the threshold) to 1 (above the threshold). On the other hand, fuzzy sets can have different shapes. They can be sigmoidal, triangular, trapezoidal, Gaussian (see Figure 2), to cite a few. For a fuzzy set, the degree of membership function (μ) changes from 0 to 1. μ is the fuzzy unit of information, called “fit”. It derives that fuzzy logic is an infinite-valued logic. 

Fuzzy logic can be used to describe any non-linear cause and effect relationship by building a fuzzy logic system (FLS). The construction of an FLS requires three fundamental steps. First, the granulation of all the variables in fuzzy sets. The number, position, and shape of the fuzzy sets are context-dependent. Second, the graduation of all the variables. A word, often an adjective, labels every fuzzy set. Third, the relationships between input and output fuzzy sets are described through syllogistic statements of the type “IF…, THEN….”, called fuzzy rules. The “IF…” part is the antecedent and involves the linguistic labels chosen for the input fuzzy sets. The “THEN…” part is the consequent and involves the linguistic labels chosen for the output fuzzy sets.

**Figure 3:**
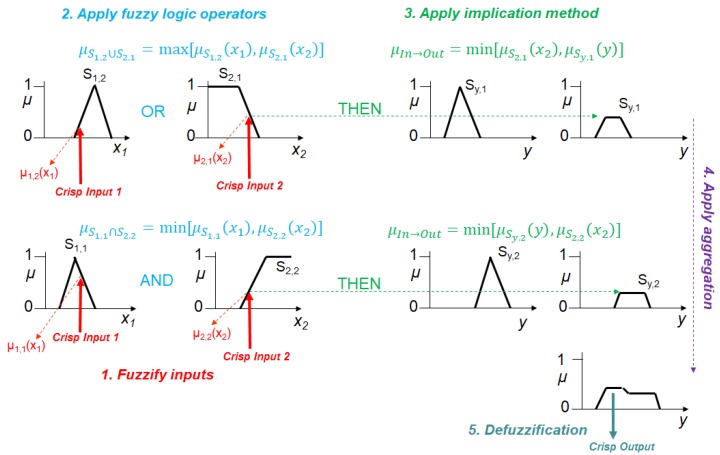
The flow of information in a fuzzy logic system where AND, OR and the implication have been implemented through the minimum, the maximum, and the minimum operators, respectively.

When we have multiple inputs, these are connected through the AND, OR, NOT operators [12]. AND corresponds to the intersection (e.g., the intersection of two fuzzy sets, whose membership functions are $\mu_{S_{1}}$ and $\mu_{S_{2}}$, can be $\mu_{S_{1}\cap S_{2}}=\min\left[\mu_{S_{1}},\mu_{S_{2}}\right]$ or $\mu_{S_{1}\cap S_{2}}=\mu_{S_{1}}\times \mu_{S_{2}}$); OR corresponds to the union (e.g., the union of the two sets S_1_ and S_2_ can be $\mu_{S_{1}\cup S_{2}}=\max\left[\mu_{S_{1}},\mu_{S_{2}}\right]$ or $\mu_{S_{1}\cup S_{2}}=\mu_{S_{1}}+\mu_{S_{2}}-\mu_{S_{1}}\times \mu_{S_{2}}$); NOT corresponds to the complement (e.g., the membership function for the Fuzzy complement of S is $\mu_{\bar{S}}=1-\mu_{s}$). Fuzzy rules may be provided by experts or can be extracted from numerical data. After the granulation, the graduation of all the input and output variables, and the formulation of the fuzzy rules, we have a FLS that is a predictive tool or a decision support system for the particular phenomenon it describes. The way an FLS works is schematically depicted through an example in Figure 3. 

The information flows along the path traced by the arrows. First, the two crisp inputs are transformed in degrees of membership to the input fuzzy sets. This step is the so-called fuzzification process. It turns on all the fuzzy rules that involve the input Fuzzy sets “activated” by the crisp inputs. Second, the logic operators (AND, OR in Figure 3) combine the degrees of membership of the input fuzzy sets belonging to the two input variables. Third, the fuzzy implication method transforms the output fuzzy sets of each activated fuzzy rule through either the minimum or the product operator (in Figure 3, the minimum operator is used). Fourth, the activated output fuzzy sets are in turn aggregated through the maximum operator. Finally, the defuzzification procedure coverts the output Fuzzy sets in a crisp output value. The defuzzification method can be “the mean of the maxima”, “the centroid”, and others (for more information, see the tutorial by Mendel [12]). In a control-system application, the crisp output corresponds to a control action. In a signal processing application, such a number corresponds to a forecast or the location of a target. Fuzzy logic systems with adaptive capabilities are also used to predict chaotic time series [13,14]. The Fuzzy logic rules work as patches covering the chaotic attractors in their phase space. The rules are established through a learning procedure requiring a training data set.

The simulation and analysis of the dynamics of complex systems can be accomplished by the fuzzy cognitive maps (FCMs) [15]. The FCMs are an extension of the cognitive maps introduced by Axelrod [16]. An FCM is a graph, which consists of nodes and edges. The nodes represent concepts relevant to a given complex system, and edges represent the causal relationships among the nodes. Each edge is associated with a number that determines the degree of causal relation. The strengths of the relationships are usually normalized to the [−1, +1] range. Value of −1 is full negative, +1 full positive, and 0 denotes no causal effect. The structure of an FCM is represented by a square matrix, called connection matrix, which reports all the weight values for edges between corresponding concepts represented by rows and columns. A complex system with *n* nodes will be represented by *n* × *n* connection matrix. The prediction of the evolution of a complex system is carried out after assigning (I) a vector of initial values to the states of the nodes and (II) a function that transforms the product of the connection matrix with the vector of the initial values into a vector representing the values of the nodes at an instant later. The transformation function can be discrete (such as the Heaviside function) or continuous (such as the logistic function). In the case of discrete functions, the complex systems can evolve into an attractor constituted by a stable node or limit cycle. In the case of continuous functions, even strange attractor can emerge [17].

**Figure 4:**
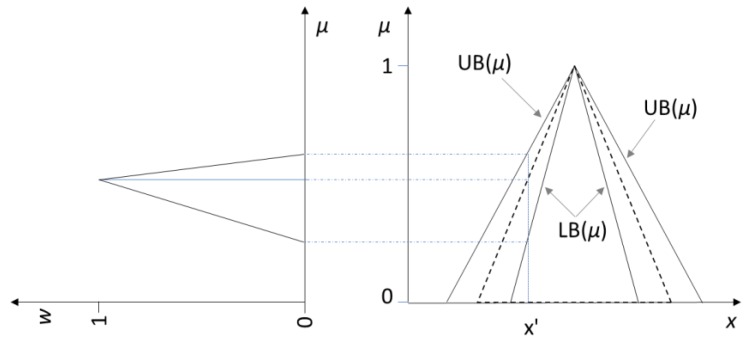
An example of type-2 fuzzy set. The original type-1 fuzzy set is the dashed triangular set. The lower (LB) and upper (UB) bounds define the footprint of uncertainty. The plot on the left shows the trend of the secondary membership (*w*) when x=x′

Both fuzzy logic systems and fuzzy cognitive maps can be built either by human experts or automatically through learning algorithms. It may happen that the membership functions of the fuzzy sets are not certain but have definite degrees of uncertainty. For these cases, Zadeh introduced [18] the concept of type-2 fuzzy sets that is an extension of the concept of an ordinary fuzzy set, i.e., a type-1 fuzzy set. Type-2 fuzzy sets have grades of membership that are themselves fuzzy. At each value of the primary variable *x*, the membership is a function and not just a point value: it is the secondary membership function (*w*). The domain of *w* is in the interval [0, 1] and its range is also in [0, 1] (see Figure 4). Therefore, the membership function of a type-2 fuzzy set is three-dimensional [19]. If projected on a plane, it gives rise to the footprint of uncertainty, which is bound by a lower membership function (LB) and an upper membership function (UB). In Figure 4, LB and UB are represented as continuous black lines. The footprint of uncertainty embeds the type-1 fuzzy set delimited by dashed lines. Type-2 fuzzy sets find many applications in intelligent control, pattern recognition, intelligent manufacturing, time series prediction, and other fields [20].

.

## 3. Fuzzy Logic and the Human Nervous System

Fuzzy logic is a valid model of the human capability to compute with words because there are structural and functional analogies between the human nervous system (HNS) and a Fuzzy logic system [21,22]. The HNS is a complex network of billions of nerve cells distributed throughout our organism [23]. It monitors the environment and our body, and it masters our behavior after collecting information, processing it, taking decisions. The HNS comprises three elements: (I) the sensory system; (II) the central nervous system; (III) the effectors’ system. The sensory system catches physical and chemical signals and transduces them in electrochemical information that is sent to the brain. Into the brain, information is integrated, stored and processed. The outputs of the cerebral computations are electro-chemical commands sent to the components of the effectors’ system, i.e., glands and muscles. Our sensory system encompasses eight sensory subsystems: a visual system to detect light; an olfactory and a gustatory system to probe chemicals in the air we breathe and in what we uptake through our mouth, respectively; an auditory, tactile, and proprioceptive system provided with mechanoreceptors that perceive either steady or vibrating or instantaneous mechanical forces; thermoreceptors to distinguish cold from warm stimuli; nociceptors to alert our body in the presence of noxious situations. Each sensory subsystem has a hierarchical structure. At the lowest level, there is a collection of receptor proteins. At an upper level, there are receptor cells that contain several replicas of the receptor molecules. We have many copies of the receptor cells properly distributed in space, often covering a tissue. The tissue may be located in an organ provided with an accessory structure that conveys the stimuli to the receptor cells. Every sensory subsystem encodes four aspects of a stimulus: its modality (M), intensity ($I_{M}$), spatial distribution ($I_{M}\left(x,y,z\right)$), and time evolution ($I_{M}\left(t\right)$). This multiple information is encoded hierarchically. In fact, the modality is encoded at the molecular level. The ensemble of the molecular receptors of a specific sensory subsystem works as a collection of molecular fuzzy sets: they granulate the modality of the kind of stimulus they sense. Signals that are perceived by the same sensory subsystem but have distinct modalities belong to the collection of the molecular fuzzy sets at different degrees. In other words, the modality of the signals is encoded as fuzzy information at the molecular level through the molecular Fuzzy sets that work in parallel. 

**Figure 5:**
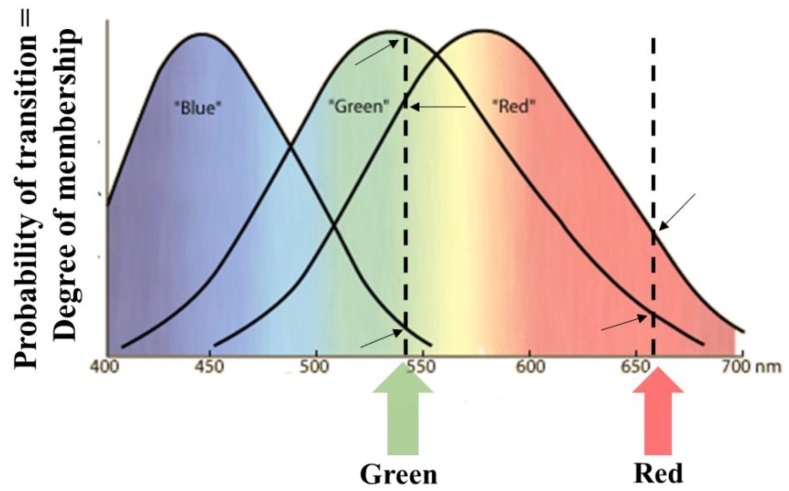
Absorption spectra of the “Blue”, “Green”, and “Red” photoreceptors that partition the visible spectral region in three fuzzy sets. Beams having different colors belong to the three molecular Fuzzy sets at different degrees. The degrees of membership of one pure green and one pure red beam to three Fuzzy sets are shown (see the arrows).

An example is shown in Figure 5. It regards our visual system. The modality is the spectral composition of the light. We have three types of photoreceptor proteins, labeled as “Blue”, “Green”, and “Red”, respectively. They allow us to distinguish colors. Their absorption spectra granulate the visible spectral region in three molecular fuzzy sets. Each band is due to the vibrational energies of the lowest excited $\pi^{*}$ state of the retinal chromophore. Light beams having distinct spectral compositions belong to the three molecular fuzzy sets at different degrees (in Figure 5, the memberships of a green and a red light are depicted).

**Figure 6:**
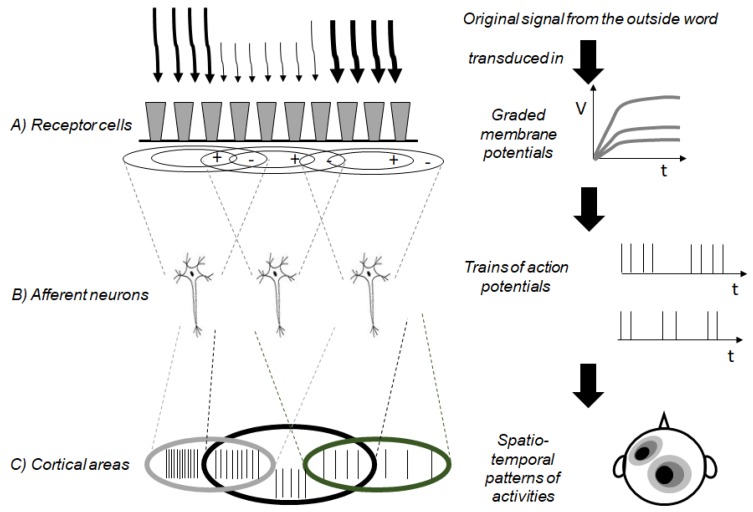
Scheme of the action of a sensory subsystem made of three principal elements described as three collections of fuzzy sets. First, the sensory cellular Fuzzy sets (**A**) that encode the information of a signal as graded potentials. Second, the afferent neurons (**B**) whose receptive fields are fuzzy sets: they encode the information as firing rates of the action potential trains. Third, the cortical areas (**C**) that are partitioned in different dynamic regimes giving rise to an infrastructure of fuzzy sets encoding distinct syntactic and semantic attributes of the original signals.

In living cells, when a stimulus actively interplays with a molecular receptor that is a protein, it promotes its structural change. Within cells, there are several copies of the molecular sensors (see Figure 6A). The number of molecular receptors that are activated in a cell depends on the intensity of the stimulus. Each cell plays like a cellular fuzzy set, and the degree of membership of a stimulus to a cellular fuzzy set encodes the intensity of the stimulus. The molecular structural modifications induced by the stimulus trigger intracellular cascade reactions, finally modifying the electrochemical permeability of the receptor cells membranes. The extent of the change in the electrochemical permeability depends on how many molecular receptors have changed their structure and hence on the intensity of the stimuli. The receptor cells produce graded potentials that are analog signals. The information of such signals is usually converted in the firing rate of the action potential trains. Often, the action potentials are produced by an architecture of afferent neurons that integrate the information regarding the spatial distribution of the stimuli (see Figure 6B). In fact, every afferent neuron has a receptive field that works as a fuzzy set encompassing specific receptor cells. For instance, in the visual subsystem, the photoreceptor cells are granulated by the bipolar cells. Light shining on the center of a bipolar cell’s receptive field and light shining on its surround produce opposite changes in the cell’s membrane potential. The purpose of the bipolar fuzzy sets is to improve the contrast and definition of the visual stimuli. The center-surround structure of the receptive fields of the bipolar cells is transmitted to the ganglion cells. The accentuation of contrasts by the center-surround receptive fields of the bipolar cells is thereby preserved and passed on to the ganglion cells. The presence of overlapping receptive fields (like overlapping fuzzy sets) allows processing the information of a light stimulus in parallel and increasing the acuity by highlighting the contrasts in space and time. The action potentials generated by the afferent neurons are the ideal code for sending the information up to the brain. In the cerebral cortex, there are areas having different intrinsic rhythms [24,25,26]. They form a neural dynamic space partitioned in overlapped cortical fuzzy compartments (see Figure 6C). Such cortical fuzzy sets are activated at different degrees by separate attributes of the perceptions and produce a meaningful experience of the external and internal worlds.

Based on this description, it might seem that sensory perception is objective, universal, reproducible, and deterministic. However, this is not the case. In fact, sensory perception depends on the physiological state of the perceiver, his/her past experiences, and each sensory system is unique and not universal. Moreover, every human brain must deal with the uncertainty in the perception. Under uncertainty, an efficient way of performing tasks is to represent knowledge with probability distributions and acquire new knowledge by following the rules of the probabilistic inference [27,28]. Therefore, it is reasonable to assume that the human brain performs probabilistic reasoning, and the human perception can be described as a subjective process of Bayesian probabilistic inference [29,30]. In fact, the frequentist probability can be used only in the case of a large number of trials. According to the Bayesian probabilistic inference, the perception of a signal $I_{M}\left(x,y,z,t\right)$ by cortical cells $CC_{M}$ is given by the “posterior probability” $p(I_{M}|CC_{M})$:

(1) $$p\left(I_{M}|CC_{M}\right)=\frac{p\left(CC_{M}|I_{M}\right)p\left(I_{M}\right)\;}{p\left(CC_{M}\right)},$$

**Figure 7:**
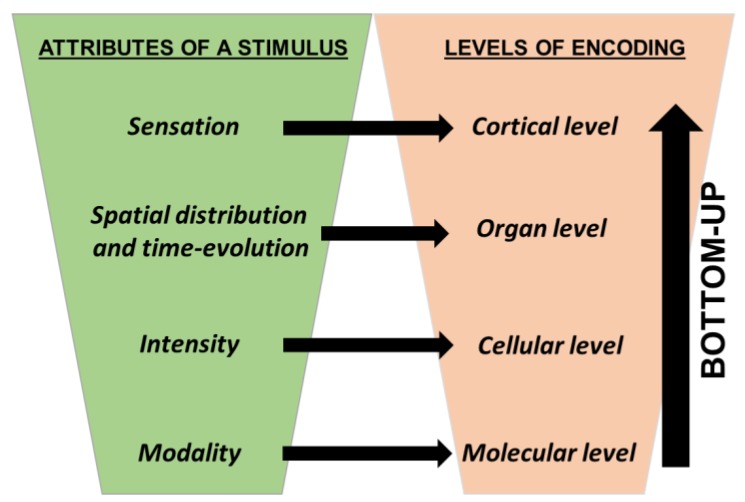
Hierarchical mechanism of encoding the information of a stimulus.

In (1), $p\left(CC_{M}|I_{M}\right)$ is the “likelihood”, $p\left(I_{M}\right)$ is the “prior probability”, and $p\left(CC_{M}\right)$ is the “plausibility”. The plausibility is only a normalization factor. In agreement with the theory of Bayesian probabilistic inference generalized in fuzzy context [31], the likelihood may be identified with the hierarchical and deterministic fuzzy information described previously in this paragraph (see also Figure 7). The prior probability $p\left(I_{M}\right)$ comes from the knowledge of the regularities of the signals and represents the influence of the brain on human perception. In fact, human perception is a trade-off between the likelihood and the prior probability [32]. If the likelihood represents the deterministic and objective part of the human perception, on the other hand, the prior probability represents its subjective contribution. The noisier and ambiguous are the features of a signal, the more prior probability driven will be the perception, and the less reproducible and universal will be the sensation. 

Sometimes, we receive multimodal signals that interact with more than one sensory subsystem. Each activated sensory subsystem produces its own mono-sensory fuzzy information. Physiological and behavioral experiments have shown that the brain integrates the mono-sensory perceptions to generate the final sensation [33]. Multisensory processing pieces signals of different modality if stimuli fall on the same or adjacent receptive fields (according to the “spatial rule”) and within close temporal proximity (according to the “temporal rule”). Since sensory modalities are not equally reliable, and their reliability can change with context, multisensory integration involves statistical issues, and it is often assumed to be a Bayesian probabilistic inference [34]. Clearly, the experience of the world is influenced by the past perceptive events, stored in the memory presumably under the shape of fuzzy rules. These stored events and rules confer to the humans the remarkable power of making decisions in complex situations and recognizing variable patterns. 

## 4. The Methodologies to Implement Fuzzy Sets and Process Fuzzy Logic at the Molecular Level

Fuzzy logic is routinely implemented in digital electronic circuits. However, the best accomplishments of FLSs have been achieved through analog electronic circuits. Whereas the digital circuits are based on electrical signals that vary steeply in sigmoid manner, the analog circuits are based on signals that vary smoothly in hyperbolic or linear manner. The analog circuits guarantee the best implementations of an infinite-valued logic that is fuzzy logic. 

In the recent years, fuzzy logic has been implemented by using even molecules and chemical reactions. Three principal strategies can be outlined: 

The first strategy is an imitation of the sensory subsystems described in the previous paragraph. In every sensory subsystem, there is a collection of distinct sensory cells that works as an ensemble of cellular fuzzy sets embedding molecular fuzzy sets. The cellular fuzzy sets work in parallel. The information of a stimulus is encoded as a vector of degrees of membership of the stimulus to the cellular fuzzy sets. This strategy will be called the “fuzzy parallelism” approach.

The second strategy is an imitation of how the proteins work in the immune and the biomolecular information systems. Almost every protein is a fuzzy set because it exists as an ensemble of many conformers that have context-dependent dynamic behavior. The macromolecular conformers are adaptable and subjected to the laws of the natural selection. They are the “words” of the cellular language. The imitation of the proteins of the cells and the immune system allows to implement the so-called “conformational fuzziness” strategy. 

Finally, the third strategy derives from the fuzziness of the quantum world and it will be called “quantum fuzziness”. When superimposed quantum states undergo decoherent phenomena, it is possible to exploit heaps of molecules to process fuzzy logic through macroscopic, smooth, analog input and output variables. 

Examples of the three strategies are described in the following three subparagraphs. 

### 4.1. The “Fuzzy Parallelism” Approach

**Figure 8:**
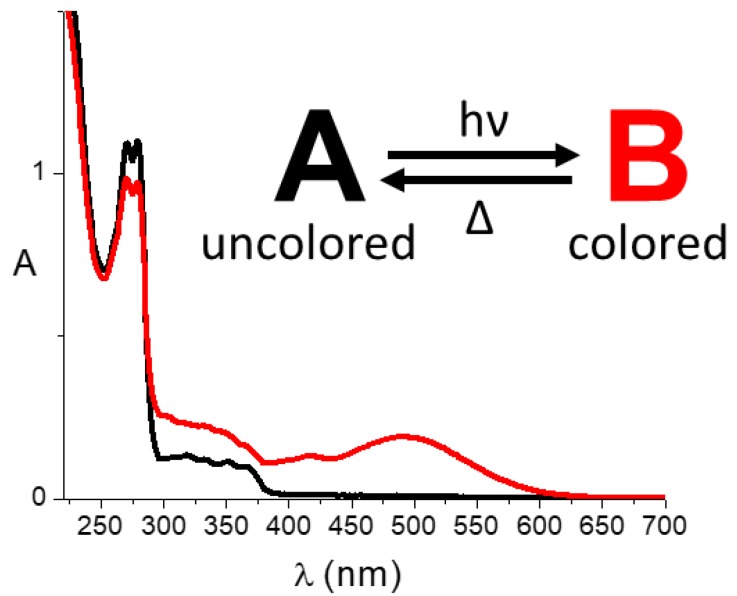
Example of a direct thermally reversible photochromic compound.

In Section 3, we have discovered that the absorption bands of the three photoreceptor proteins present on the fovea of the retina play as three molecular fuzzy sets. Lights that differ in their spectral compositions belong to the three bands at distinct degrees, and they are perceived as different colors. Moreover, the millions of replicas of the three photoreceptor proteins within each photoreceptor cell allow determining the intensity of the signals at every wavelength. The imitation of the way we distinguish colors has allowed the design and implementation of chemical systems that extend human vision to the UV [35,36]. Such chemical systems are based on direct thermally reversible photochromic compounds. A thermally reversible photochromic compound is a species that in the absence of any radiation, it exists in a structure (i.e., A in Figure 8) that absorbs just in the UV and it is uncolored. Upon UV, it transforms in B that also absorbs in the visible region. When B is formed, the system becomes colored (see Figure 8). The transformation of A into B is thermally reversible. In other words, if we discontinue the UV irradiation, the color bleaches because the B molecules transform back to the original structure A, spontaneously at room temperature. Mixtures of properly chosen direct thermally reversible photochromic compounds extend the human capability of distinguishing electromagnetic spectra to the UV region. Such mixtures, called biologically inspired photochromic fuzzy logic (BIPFUL) systems, are designed by the following procedure. First, the absorption bands of the uncolored forms, *A_i_*, are assumed to be input fuzzy sets. Second, the absorption bands of the colored forms, *B_i_*, are assumed to be output fuzzy sets. Third, the algorithm expressing the degree of membership of the UV radiation, having intensity $I_{0}\left(\lambda_{irr}\right)$ at the wavelength $\lambda_{irr}$, to the absorption band of the *A_i_* compound is:

(2) $$\mu_{UV,A_{i}}=\Phi_{PC,A_{i}}\left(\lambda_{irr}\right)I_{0}\left({}_{irr}\right)\left(1-10^{-\varepsilon_{A_{i}}C_{0,i}l}\right),$$

In Equation (2), $\Phi_{PC,A_{i}}(\lambda_{irr}$) is the photochemical quantum yield of photo-coloration for *A_i_*, $\varepsilon_{A_{i}}$ is the absorption coefficient at ${}_{irr}$ for the *A_i_* photochromic species, and $C_{0,i}$ is its analytical concentration. Finally, the equation expressing the activation of the *B_i_* output fuzzy sets is:

(3) $$A_{B_{i}}=\frac{\varepsilon_{B_{i}}\left(\lambda_{an}\right)}{k_{\Delta ,i}}\mu_{UV,A_{i}}.$$

In Equation (3), $A_{B_{i}}$ is the absorbance at the wavelength $\lambda_{an}$ into the visible and due to the coloured form of the *i*-th photochromic species; $\varepsilon_{B_{i}}\left(\lambda_{an}\right)$ is its absorption coefficient, and $k_{\Delta ,i}$ is the kinetic constant of the bleaching reaction for *B_i_*. Each absorption spectrum recorded at the photo-stationary state will be the sum of as many terms represented by equation (3) as there are photochromic components within the BIPFUL system. The BIPFUL systems that have been devised are made of naphthopyrans and spiroxazines, and they allow to discriminate the three regions of the UV spectrum, i.e., UV-A, UV-A, UV-B, and UV-C. 

The imitation of all the other sensory subsystems, conceived as hierarchical fuzzy systems where a collection of distinct molecular and cellular fuzzy sets work in parallel (see Section 3), should allow to devise artificial sensory systems that have the power of extracting the essential features of stimuli and recognizing variable patterns.

### 4.2. “Conformational Fuzziness”

Within every living cell, there are many proteins that work as if they were the neurons of the “cellular nervous system”. They participate in the signaling and genetic networks and allow the cell to respond to the ever-changing environmental conditions. Specific proteins, called antibodies, are also the fundamental ingredients of the immune system that protects our bodies from intruders. A limited set of flexible antibodies can bind a wide range of antigens. Proteins are ubiquitous in living beings and they play multiple roles, due to their “dynamism and evolvability” [37]. In fact, proteins are conformationally dynamic and exhibit functional promiscuity. Conformational dynamism and heterogeneity enable context-specific functions to emerge in response to changing environmental conditions and, furthermore, allow a single structural motif to be used in multiple settings [38]. The conformational flexibility and heterogeneity of proteins represent their fuzziness. 

Conformational fuzziness is not a prerogative feature of proteins. Even the long polymer of chromatin in the nucleus of eukaryotic cells is Fuzzy. Some portions contain heterochromatin made of DNA packed tightly around histones. Some other areas contain euchromatin that is DNA loosely packed. Usually, genes in euchromatin are active, whereas those in heterochromatin are inactive. Euchromatin exposes a broader and rougher surface to the proteins scanning for their target sequences. Heterochromatin is flatter, smoother, and with a less extended surface [39]. Chromatin organization is highly dynamic, varying both during the cell cycle and among different cell types [40]. 

**Figure 9:**
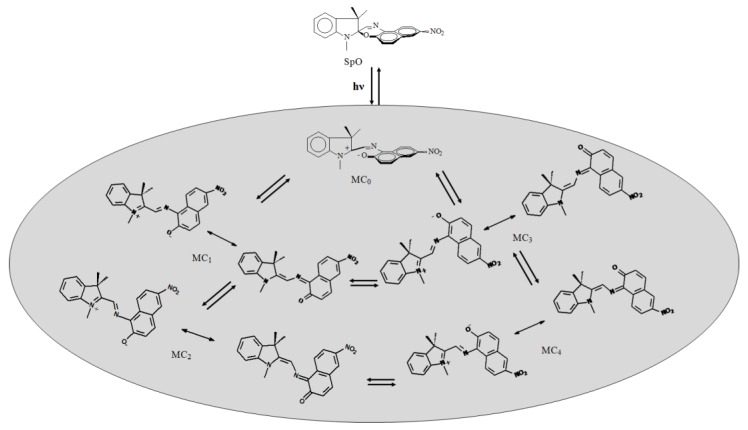
Just of a few of all the possible conformers of a merocyanine (MC_i_) produced by irradiation of a spirooxazine (SpO).

Conformational fuzziness is not unique to macromolecules, but it can be experienced even with simple molecules. An example is the fuzziness of the merocyanine (MC) that is generated by UV irradiation of the spirooxazine (SpO) shown in Figure 9 [41]. Since MC has a flexible molecular skeleton, it gives rise to many conformers. The number and type of conformers depend on the physical and chemical context (for example, temperature, solvent, and the presence of a docking glycine).

Whatever the compound is, being either a macromolecule or a molecule, the ensemble of its conformers plays like a molecular Fuzzy set. Its fuzziness may be quantified by determining its fuzzy entropy. A definition of fuzzy entropy based on Shannon’s function of information entropy is [42,43]:

(4) $$H=-K\sum_{i=1}^{n}(\mu_{i}\log_{10}\left(\mu_{i}\right)+\left(1-\mu_{i}\right)\log_{10}\left(1-\mu_{i}\right)),$$

where $\mu_{i}$ is the relative weight of the *i*-th conformer, n is the total number of conformers, and K=(1/n) is a normalization factor. The fuzzy entropy of a compound is context-dependent, like the meaning of a word in natural language. In fact, conformationally heterogeneous structures are adaptable to many different contexts. Of course, the fuzzy entropy of a macromolecule is significantly larger than that of a simple molecule. Among proteins, those completely or partially disordered [44] are the fuzziest. Their pronounced fuzziness makes them multifunctional and even able to moonlight [45], i.e., play distinct functions, depending on their context. 

### 4.3. “Quantum Fuzziness”

**Figure 10:**
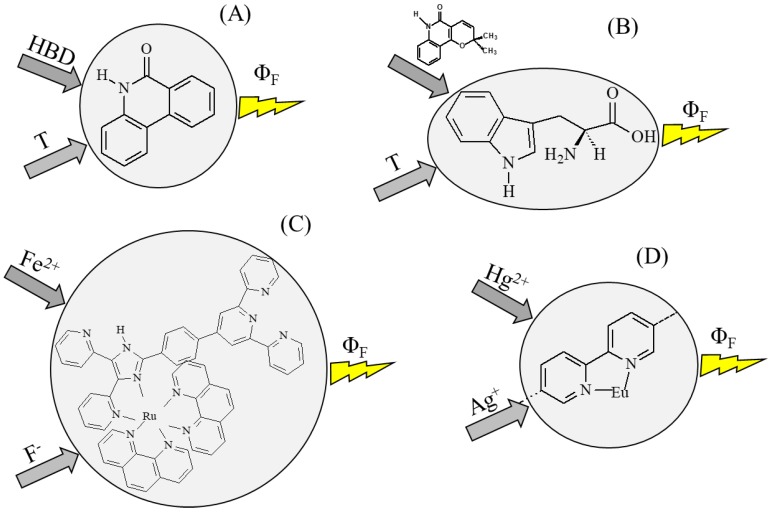
Dependence of the fluorescence quantum yield of 6(5*H*)-phenanthridinone (**A**), tryptophan (**B**), a ruthenium complex (**C**), and europium bounded to a metal-organic framework (**D**) on physical and chemical inputs.

Isolated microscopic systems exist in a superposition of states. For instance, if there are two accessible states, indicated as |0〉 and |1〉, the isolated microscopic system exists in a quantum state |Ψ〉 that is a linear combination of |0〉 and |1〉:

(5) |Ψ〉=a|0〉+b|1〉,

where a and b are complex numbers that verify the normalization condition ${\left|a\right|}^{2}+{\left|b\right|}^{2}=1$. The states |0〉 and |1〉 can be imagined as two fuzzy sets. Their vagueness, i.e., their fuzziness is outlined by the Heisenberg’s Uncertainty Principle. The |Ψ〉 state belongs to |0〉 and |1〉 with degrees that are ${\left|a\right|}^{2}$ and ${\left|b\right|}^{2}$, respectively. |Ψ〉 is a qubit, i.e., the elementary unit of the quantum information. The qubit can be described as a unit vector in a two-dimensional Hilbert space. The state of the qubit can be also represented by the following equation:

(6) $$|\Psi \rangle =\cos\left(\frac{\theta }{2}\right)|0\rangle +e^{i\varphi }sin\left(\frac{\theta }{2}\right)|1\rangle ,$$

where θ and φ define a point on the unit three-dimensional sphere, called the Bloch sphere. Logic operations on qubits can be visualized as rotations of the unit vectors on the Bloch sphere, preserving the norm of the quantum states. If a microscopic system is a superposition of *n* qubits, it has 2*^n^* accessible states, simultaneously. If we make an operation on this system, we manipulate 2*^n^* states, at the same time. Therefore, it is evident the alluring computational power of quantum logic. However, the main difficulty is to avoid the decoherence of the superimposed quantum states, which can be induced by deleterious interactions with the surrounding environment [46]. The decoherence induces the collapse of a qubit in one of its two originally accessible states, either |0〉 or |1〉, with probabilities ${\left|a\right|}^{2}$ and ${\left|b\right|}^{2}$, respectively. Whenever the decoherence is unavoidable, the single particles can be used to process discrete logics, i.e., binary or multi-valued crisp logics [47,48]. Of course, specific microscopic techniques, reaching the atomic resolution, are needed to carry out the computations. Alternatively, we may think of making computations by exploiting large assemblies of particles, e.g., molecules. Vast collections of molecules (amounting to the order of the Avogadro’s number) appear as bulky materials. The inputs and outputs for making computations become macroscopic variables that can change in a continuous manner. The relations establishing between the inputs and the outputs can be either steep or smooth. Steep, sigmoid functions are suitable to implement discrete logics, whereas both linear and nonlinear smooth functions are suitable to build fuzzy logic systems [49]. Some fuzzy logic gates and operations have been implemented by the hybridization reaction of DNA [50,51] and the supramolecular interactions between carbohydrates and proteins [52]. Other fuzzy logic systems have been built by exploiting the dependence of the fluorescence quantum yield on physical and chemical inputs. One example is the dependence of the fluorescence of 6(5*H*)-phenanthridinone (see Figure 10A) on the hydrogen bonding donation ability of the solvent (HBD) and the temperature [53]. Another example is given by tryptophan, both as isolated molecule and bonded to the serum albumin, whose fluorescence depends on the temperature and the amount of the quencher flindersine (see Figure 10B) [54]. A further example is a ruthenium complex, whose fluorescence depends on Fe^2+^ and F^−^ (see Figure 10C) [55]. A final example is the fluorescence of europium bound to a metal-organic framework, which depends on metal cations, such as Hg^2+^ and Ag^+^ (see Figure 10D) [56]. The emission of light is a preferable output because it bridges the gap between the microscopic and the macroscopic world. A multi-responsive chromogenic compound, belonging to the class of spirooxazine, has been used for the implementation of the all fundamental fuzzy logic gates, AND, OR, and NOT [57]. The protons, Cu^2+^, and Al^3+^ ions were used as inputs, and the color coordinates (R, G, B) or the colorability [41] of the chromogenic compound as outputs. Then, other platforms have been proposed. For example, a multi-state tantalum oxide memristive device [58] and an anthraquinone-modified titanium dioxide electrode [59]. Even, the Belousov-Zhabotinsky reaction, carried out in oscillatory regime and in an open system [60], allows to implement all the fundamental fuzzy logic gates by using bromide and silver ions as chemical inputs and the period of the oscillations as outputs. Finally, the “hydrodynamic photochemical oscillator”, which is a thermally reversible photochromic compound combined with the convective motion of the solvent, is suitable to implement fuzzy logic systems when it works in chaotic regime [61]. All these examples show that fuzzy logic can be processed not only by conventional electronic circuits but also by unconventional chemical systems exhibiting analog input-output relationships in either the liquid or the solid phase. 

## 5. Perspectives of the Fuzziness of the Molecular World

Fuzzy logic is a valid model of the human power to compute with words and take decisions in complex situations. The closer one looks at the real-world problems, the fuzzier become their solutions. Fuzzy logic is playing a relevant role in the field of artificial intelligence when we deal with complex systems. 

This work highlights that even the molecular world is fuzzy. In fact, quantum logic is fuzzy (“quantum fuzziness”). A qubit is a superposition of two distinct quantum states that are like fuzzy sets. Therefore, quantum logic might be considered a particular kind of fuzzy logic. When decoherent phenomena induce the collapse of qubits, it is not possible to process quantum logic. However, by working with large collections of molecules, it is feasible to implement fuzzy logic systems, when causal, macroscopic, smooth, analog input-output relationships are found. 

In the human sensory system, the sensory cells that are fuzzy sets, containing molecular fuzzy sets, collect a large amount of data. The hierarchical architecture of the afferent and cortical neurons, which is based on the overlapping of their receptive fields, allows extracting only the meaningful information of the big data contained in the stimuli. The imitation of the principal features of the sensory system, in particular of what we called as “fuzzy parallelism”, should allow devising artificial sensory system able to extract the essential characteristics of the complex stimuli. Hence, such artificial sensory systems should be suitable to recognize variable patterns. 

The computational power of the cells and the human immune system derives from the “conformational fuzziness” of their macromolecules. By exploiting the conformational elasticity of molecules, especially proteins, it is possible to process fuzzy logic. In fact, the “conformational fuzziness” makes molecules adaptable to their microenvironment. This feature is suitable to implement the dependence of the information on the context.

By processing fuzzy logic at the molecular level, we want to promote the development of the chemical artificial intelligence. The purpose of chemical artificial intelligence is to mimic the performances of the human intelligence by using not software or hardware, but rather chemical and photochemical reactions in wetware. In fact, there exist chemical systems that can work as surrogates of the neural dynamics [62,63,64,65]. These systems can interact and communicate by exploiting both chemical and electrical and optical signals. They are the fundamental components of a futuristic opto-/electro-brain-like computing machine that should be suitable to recognize variable patterns and compute with words. There is a long path before the concrete implementation of this new generation of computing machines, more similar to the brain rather than to the electronic computer from both the structural and the functional point of view. Further analysis of the human nervous system and further development of the theory of fuzzy logic are needed. For example, the receptive field of a neuron can inspire a new kind of fuzzy set (i.e., Type-III fuzzy set) where we distinguish inhibitory and excitatory actions. With this new kind of fuzzy set, implemented somehow artificially, the recognition of variable patterns should become easier. Moreover, the chemical artificial intelligence will boost the development of the soft robotics. Soft robots, also called “chemical robots”, will be easily miniaturized and implanted in living beings [66,67,68,69,70,71]. They will interplay with cells and organelles for biomedical applications. They will become auxiliary elements of the human immune system to defeat diseases that are still incurable. 

Finally, this field of research could give clues about the origin of the life on Earth. In fact, the appearance of the life on Earth, occurred roughly 3.5 billion of years ago, was like a “phase transition”. It was a transition from inanimate chemical systems, unable to encode, process, communicate and store information, to the living chemical systems, able to exploit the matter and energy to encode, process, send, and store information. The development of chemical artificial intelligence could unveil how that unique “phase transition” happened.
